# Mechanisms of Foot-and-Mouth Disease Virus Tropism Inferred from Differential Tissue Gene Expression

**DOI:** 10.1371/journal.pone.0064119

**Published:** 2013-05-28

**Authors:** James J. Zhu, Jonathan Arzt, Michael C. Puckette, George R. Smoliga, Juan M. Pacheco, Luis L. Rodriguez

**Affiliations:** Foreign Animal Disease Research Unit, Agricultural Research Unit, United States Department of Agriculture, Plum Island Animal Disease Research Center, Orient Point, New York, United States of America; Institut National de la Santé et de la Recherche Médicale U 872, France

## Abstract

Foot-and-mouth disease virus (FMDV) targets specific tissues for primary infection, secondary high-titer replication (e.g. foot and mouth where it causes typical vesicular lesions) and long-term persistence at some primary replication sites. Although integrin αVβ6 receptor has been identified as primary FMDV receptors in animals, their tissue distribution alone fails to explain these highly selective tropism-driven events. Thus, other molecular mechanisms must play roles in determining this tissue specificity. We hypothesized that differences in certain biological activities due to differential gene expression determine FMDV tropism and applied whole genome gene expression profiling to identify genes differentially expressed between FMDV-targeted and non-targeted tissues in terms of supporting primary infection, secondary replication including vesicular lesions, and persistence. Using statistical and bioinformatic tools to analyze the differential gene expression, we identified mechanisms that could explain FMDV tissue tropism based on its association with differential expression of integrin αVβ6 heterodimeric receptor (FMDV receptor), fibronectin (ligand of the receptor), IL-1 cytokines, death receptors and the ligands, and multiple genes in the biological pathways involved in extracellular matrix turnover and interferon signaling found in this study. Our results together with reported findings indicate that differences in (1) FMDV receptor availability and accessibility, (2) type I interferon-inducible immune response, and (3) ability to clear virus infected cells via death receptor signaling play roles in determining FMDV tissue tropism and the additional increase of high extracellular matrix turnover induced by FMDV infection, likely via triggering the signaling of highly expressed IL-1 cytokines, play a key role in the pathogenesis of vesicular lesions.

## Introduction

Foot and mouth disease (FMD) is one of the most contagious and economically devastating viral animal diseases. It is caused by FMD virus (FMDV), a positive-sense, single stranded RNA virus member of the family Picornaviridae (genus *Aphtovirus*). Susceptible hosts include several domesticated (e.g. cattle, Asian buffalo, sheep, goats, and swine) and wild (e.g. African buffalo and wild boar) cloven-hoofed animals. The morbidity is very high but the mortality is generally low in adult hosts. The infection in cattle commonly occurs via the respiratory route by aerosolized virus [1]. After infection, the virus replicates locally in primary replication sites such as the nasopharynx and lung during the pre-viremic phase [2], [3], [4], [5]. The infection then spreads via the bloodstream (viremic phase) to secondary replication sites where the virus grows to high titers and causes typical blisters, erosions, and ulcers at specific regions of the oral cavity, feet, and occasionally other sites [1]. Persistent infection can occur for long periods (30 days –5 years) with virus persisting at certain primary infection sites (e.g. nasopharynx) in a subset of infected animals [6], [7]. The mechanisms that determine FMDV tissue tropism including target sites for primary, secondary, and persistent infection remain undetermined despite extensive research on this virus discovered over 100 years ago.

Several host integrins including αvβ1, αvβ3, αvβ5, αvβ6 and αvβ8 have been shown to be receptors for FMDV in cultured cells and the mechanism of binding has been shown to involve an Arg-Gly-Asp (RGD) amino acid sequence motif present on the FMDV VP1 [8], [9], [10], [11], [12], [13], [14]. However, several lines of evidence suggest that αvβ6 is the primary FMDV receptor in animals and this receptor expression is associated with FMDV tropism [15], [16], [17], [18]. Despite this evidence, the tissue expression patterns of the integrins do not fully explain the tropism and pathogenesis as many non-susceptible tissues also express the αvβ6 integrin [18]. Results from several studies using other piconaviruses also suggest that viral receptors are not the sole determinants of virus tropism [19]. Interestingly, the site-specific location of vesicular lesions is not unique to FMDV. Vesicular stomatitis virus, swine vesicular disease virus, and vesicular exanthema of swine virus have nearly identical lesion sites and clinical signs as FMD in cattle and swine, suggesting that these targeted tissues possess certain biological characteristics rendering them susceptible to a common pathway associated with these viral vesicular diseases.

Like other pathogenic viruses, the FMDV genome encodes several proteins that facilitate evasion of the innate immune response of infected cells. For example, the leader protease (Lpro) can inhibit host cap-dependent translation by cleaving translation initiator factor eIF4G but has no effects on viral cap-independent translation [20]. Lpro is also found to enter the cell nucleus and inhibit the induction of IFNβ and some interferon stimulated genes [21], [22]. Additionally, there are several reports showing that altering some host cellular factors such as Rab5 and RNA helicase A can inhibit FMDV replication [23], [24]. These findings indicate that host factors other than the virus receptors may be contributing to FMDV tissue tropism.

Because cells differentiate by alternating their gene expression, differential tissue gene expression may provide valuable insights into the molecular mechanisms involved in determining FMDV tissue tropism. We designed a custom bovine whole genome expression microarray and used microarray analysis to identify genes differentially transcribed among tissues targeted and not targeted by FMDV for primary, secondary, and persistent infection in infected and non-infected cattle. The bioinformatic analyses of the differentially expressed genes lead to the identification of several biological processes/pathways which activity could be affected by the differential expression. Based on the expression of the genes involved in these biological processes and on relevant findings from the literature, we propose molecular bases that help explain the mechanisms of FMDV tissue tropism.

## Materials and Methods

### Animal Inoculations, Tissue Collection, and RNA Preparation

All animal studies were conducted under an approved Institutional Animal Care and Use Committee protocol. Five Holstein steers weighing 225 to 300 kg were obtained from an accredited experimental-livestock provider (Thomas-Morris Inc, Reisterstown, MD). Two of the steers were aerosol-inoculated as previously described [5] with FMDV-A24-Cruzeiro and three were mock inoculated with sterile cell culture media. All steers were euthanized at 72 hours post infection which was coincident with the first observation of generalized FMD (vesicles) in the two infected animals. Necropsies and tissue collections were performed immediately after euthanasia as previously described [3]. For the sites with observed vesicular lesion, tissues with ∼0.75 centimeter away from the vesicular lesion were sampled to avoid the impact of tissue necrosis on RNA quality. These tissues were immediately frozen in liquid nitrogen and stored in −70°C. Total tissue RNA was isolated from the frozen tissues using a Qiagen RNA isolation kit (Qiagen, Germantown, MD). The quantity and integrity of RNA samples were examined with a NanoDrop 1000 (Thermo Fisher Scientific, Waltham, MA) and a Bioanalyzer 2000 (Agilent Technologies, Santa Clara CA), respectively. Viral RNA in sera and nasal secretions was quantitated using real-time RT-PCR as previously described [3].

### Description and Classification of Analyzed Tissues

Selected tissues were collected based on pathogenesis studies as previously described [3]. The dorsal soft palate (DSP), dorsal nasal pharynx (DNP), and mid anterior lung (LNG) have been shown to be the primary replication sites (PRS) based upon consistent localization of FMDV to these tissues when collected from previremic steers infected with FMDV [3]. The nasal turbinate epithelium (NTE), previously shown not to be infected during the early phase of FMD pathogenesis, was used individually as a negative control for PRS. The interdigital cleft skin (IDC), coronary band (CB), and tongue epithelium (TE) are secondary replication sites (SRS) where the virus can replicate to high titers and cause vesicular lesions. Despite close proximity to the feet, the metacarpal skin (MCS) has never been described as a FMD lesion site in cattle and was therefore chosen as a negative control for SRS. For data analyses, MCS and NTE were also grouped as not targeted sites (NTS) based on low or no viral replication compared to their corresponding targeted tissues. DSP and DNP were also grouped as persistent infection sites (PIS) to contrast all other tissues where FMDV persistence does not occur.

### DNA Microarray Design

A bovine whole genome expression microarray was designed based on all bovine expressed sequence tags and RNA sequences from the NCBI database and assembled into unique sequences with the CAP3 program [25]. The assembled sequences were aligned to the bovine genome sequences and displayed with the UCSC genome browser [26]. Non-redundant bovine expressed sequences and sequences with homology to genes in other species but no bovine EST or RNA were selected for probe design. The custom bovine whole genome expression microarray comprised approximately 42,000 60-oligonucleaotide sense probes and was designed with ArrayDesigner 4.0 (PREMIER Biosoft International, Palo Alto CA) from the selected bovine sequences. The probes were designed with locations biased to the 3′-end of RNA sequences in order to produce high signal intensity for fluorescent labeling chemistry using poly-T priming. The annotation of the bovine microarray was based on the results of BLAST against human sequences, manual annotation based on the probe sequence alignments in the bovine genome sequence (Baylor 4.0/bosTau4 October 2007 release), and other genetic information displayed on the UCSC genome browser (http://genome.ucsc.edu/cgi-bin/hgGateway). The DNA probes for all available FMDV genome sequences were also designed and included in the array to detect FMDV RNA levels.

### Microarray Analysis

The custom bovine microarrays used in this study were manufactured by Agilent technologies. Agilent Quick Amp RNA labeling kit (Agilent Technologies, Santa Clara CA) was used for the sample labeling. Total RNA prepared from a metacarpal skin sample was labeled with Cy5 and used as a universal control for cross-array data normalization, whereas all samples including 8 tissues from each animal (40 samples) were labeled individually with Cy3. Each Cy3 labeled sample together with the control was hybrided to a microarray. The entire procedure from RNA quality check to microarray hybridization and washing was conducted using reagents and protocols provided by Agilent Technologies. The microarrays were scanned at 5 µm resolution with Cy3:Cy5 signal ratio of between 0.9 to 1 using a GenePix 4000B scanner and GenePix Pro 6.0 program (Molecular Devices, Sunnyvale, CA).

### Statistical and Bioinformatic Analyses

Microarray data were extracted from the signal intensity of Cy3 and Cy5 using GenPix Pro 6.0 program and data imported into a SQL database created with Microsoft SQL Server 2000 and Acuity® 4.0 Enterprise Microarray Informatics software (Molecular Devices, Sunnyvale, CA). The data of extracted gene expression signals were background-corrected with the average signals of nearby negative control features. All negative intensity readings were adjusted as 0. The expression data were normalized with the signal of Cy5-labeled universal control to have equal averaged signal intensity. After normalization, all expression data were added with an arbitrary small value of 1 as suggested in the EDGE program user manual (http://www.genomine.org/edge/edgehelp.pdf) to avoid statistical analysis involved with division by zero. The normalized signal data were square-root-transformed and analyzed using t-test. Differentially expressed genes (DEG) were defined as having a difference of ≥75% or 1.75 fold with a *P* value of 0.05 or smaller in all pair-wise two tissue comparisons between tissue groups. The significant differences obtained from bovine sequences without known human homologous genes were not included in the gene sets and were excluded from further analysis. Eight gene sets including up- and down-regulated in non-targeted sites (NTS) vs targeted sites (SRS+PRS), up- and down-regulated in secondary replication sites (SRS) vs other tissues (NTS+PRS), up- and down-regulated in persistent infection sites (PIS) vs others (NTS+SRS+LNG), and up- and down-regulated in primary replication sites (PRS) vs nasal turbinate epithelium comparisons were created from data obtained from three not infected animals and identified as NTS-up, NTS-down, SRS-up, SRS-down, PIS-up, PIS-down, PRS-up, and PRS-down, respectively (Table 1).

**Table 1:** The average microarray signal intensity of FMDV target sequences in the tissues of two infected animals collected at 72 hours post FMDV infection.

Infection sites	Tissues	Tissue tropism	Virus signal
Secondary replication sites	TE	High titer VR, VL, no PI	4445
	IDC		11928
	CB		871
Not targeted sites	MCS	Low VR, no VL, no PI	206
	NTE		39
Primary replication but not persistence site	LNG	Low VR, no VL, no PI	359
Primary replication and persistence sites	DSP	Low VR, no VL, PI	230
	DNP		134

CB: coronary band; DSP: distal soft palate; DSP: distal nasal pharynx; IDC: interdigital cleft skin; LNG: middle anterior lung; MCS: metacarpal skin (control for secondary replication sites); NTE: nasal turbinate (control for primary replication sites); TE: tongue epithelium; VR: FMDV replication; VL: vesicular lesion; PI: persistent infection.

The gene sets were submitted to a BIOBASE web site (http://explain30.biobase-international.com/cgi-bin/biobase/ExPlain_3.0/bin/start.cgi) using a subscribed account to identify the pathways/networks that can regulate the differential expression by analyzing over-represented transcription factor binding sites in the promoter sequences of the identified DEG and can be significantly affected by the differential gene expression using ExPlain™ program (BIOBASE, Wolfenbüttel, Germany). To identify over-represented transcription factor binding sites, all bovine gene sequences used to design the microarray probes were mapped to human genes based on the result of BLAST or annotation from the bovine genome sequence displayed in the UCSC genome browser. The promoter sequences in −500 and +100 bases of transcription starting sites of human genes homologous to the identified bovine DEG were compared to a human house-keeping gene set provided by BIOBASE as a control. The over-represented transcription factor binding sites not shared in compared gene sets were considered as the sites of interests, and signal transduction pathways that can activate transcription factors to bind to these sites are considered to regulate the differential expression. To detect biological networks that may be significantly affected by the differential gene expressions, the gene sets were analyzed to identify those with significant more DEG than that may occur randomly (false discover rate ≤0.05) using statistical tests included in the ExPlain™ program. Both up- and down-stream networks of key nodes were analysis to identify DEG regulating and regulated by the key nodes, respectively.

### Biological Inferences

Once candidate pathways/networks were detected, all genes known to be involved in these biological processes were identified and their expression levels were examined in terms of up- or down-regulation and the magnitudes of the differences between tissue groups. Significant differences (*P* = or <0.05) were analyzed with t-test. Biological impact of differential gene expression was evaluated based on known biological functions of the genes with an assumption that higher transcription levels are associated with higher biological activity of the genes. The tissue activity of indentified pathways/networks was determined based on the expression levels and functions of the genes in the pathways. Biological inferences were made according to the impact of predicted differences in the activity of the pathways on FMDV infection. The data from three non-infected and two infected animals were used to make the inferences.

### Estimate of Integrin αVβ6 Receptor Level

RGD-binding integrins were selected based on Hynes [27], which include 1 α subunit (ITGAV) and 5 β subunits (ITGB1, ITGB3, ITGB5, ITGB6, and ITGB8). According to Hynes [27], ITGB1 also forms heterodimeric receptors with ITGA1, ITGA2, ITGA3, ITGA4, ITGA6, ITGA7, ITGA9, ITGA10, and ITGA11, and ITGB3 also binds to ITGA2B. The portion of ITGB1 that may binds to ITGAV (ITGB1_v) was estimated by multiplying the signal intensity of ITGB1 with the percentage of ITGAV intensity in the total intensity of all ITGB1-binding α subunits. Similarly, the portion of ITGB3 binding to ITGAV (ITGB3_v) was calculated by multiplying the level of ITGB3 with the percentage of ITGAV level in the sum of ITGAV and ITGA2B. Finally, the expression level of integrins αVβ6 was estimated based on the product of ITGAV and the percentage of ITGB6 in the sum of ITGB1_v, ITGB3_v, ITGB5, ITGB6, and ITGB8.

## Results

### Clinical Observations

Steers inoculated with sterile media did not develop fever, vesicular lesions, or any other signs of disease or complications from the aerosolization procedure. The two steers inoculated with FMDV-A24 had very similar clinical syndromes including fever (rectal temperature >39.5C) at 2–3 days post infection and vesicles in the mouth and/or feet detected at 3 days post infection indicating that both animals were at the start of generalized FMDV infection when euthanized for tissue collection. Steer #1 had vesicles in the interdigital cleft of one foot and in the tongue, whereas steer #2 had vesicles on all 4 interdigital clefts. No other lesions were identified. Both animals were subsequently confirmed to have FMDV and viral RNA (8.36–9.08 log10 viral RNA copies/ml) in sera (viremia) and in nasal secretions at the time of euthanasia.

### Signal Intensity of FMDV Target Sequences

Varying signal intensities of FMDV microarray features were observed in the infected tissues (Table 1), whereas the tissues of the non-infected animals displayed signal intensities nearly identical to the negative control. As expected, the signal intensities of FMDV RNA from the infected secondary replication sites (sampled away from vesicular lesions) were the highest among the tissues tested. There were also differences among the SRS tissues with significantly lower signals in the coronary band than other secondary replication tissues. Not targeted tissues such as the metacarpal skin and nasal turbinate epithelium displayed 3–4 fold lower FMDV RNA signal than their corresponding targeted tissues. The signal intensity of the metacarpal skin was more than 5 times higher than that of nasal turbinate epithelium. The interdigital cleft skin and lung had the highest signal intensity of the SRS and PRS, respectively; however, the viral RNA signal intensity in the interdigital cleft skin was over 33-fold higher than that in the lung. These results are consistent with viral titers and tissue distribution observed in infected animals in previous studies [2].

### Differentially Expressed Genes

Whole genome gene expression profiles of the tissues from the three naïve animals were compared across four tissue categories selected according to their classification as not targeted sites, primary infection, secondary replication, or persistence sites. Tissue categories were based upon previously published studies describing tissue-specific FMDV loads as stated earlier. Eight gene sets were created from the tissue comparisons based on the statistical analysis described earlier (Table 2). In the tissue comparison of not targeted sites versus others, most of the DEG were up-regulated (n = 36) and only two genes were down-regulated (Table 2). Similarly, when comparing the persistent infection site versus other tissues, there were 73 up-regulated and only 4 down-regulated genes. When the SRS or PRS were compared to the rest of the tissues, there were 412 and 220 up-regulated and 103 and 360 down-regulated genes in the secondary and primary replication sites, respectively. These gene sets were statistically analyzed using the ExPlain™ program to infer the biological pathways that could be potentially involved in determining FMDV tissue tropism.

**Table 2:** The number of genes differentially expressed between tissues different in FMDV tropism in non-infected animals.

Gene set	Pair-wise tissue comparisons	Number of genes
NTS-up	MCS>IDC, TE, and CB	90
	NTE>DNP, DSP, and LNG	
NTS-down	MCS<IDC, TE, and CB,	2
	NTE<DNP, DSP, and LNG	
SRS-up	IDC>DNP, DSP, LNG, MCS, and NTE	103
	TE>DNP, DSP, LNG, MCS, and NTE	
	CB>DNP, DSP, LNG, MCS, and NTE	
SRS-down	IDC>DNP, DSP, LNG, MCS, and NTE	412
	TE>DNP, DSP, LNG, MCS, and NTE	
	CB>DNP, DSP, LNG, MCS, and NTE	
PIS-up	DNP> LNG, and NTE	73
	DSP> LNG, and NTE	
PIS-down	DNP< LNG, and NTE	4
	DSP< LNG, and NTE	
PRS-up	NTE<DNP, DSP, and LNG	220
PRS-down	NTE>DNP, DSP, and LNG	360

CB: coronary band; DSP: distal soft palate; DSP: distal nasal pharynx; IDC: interdigital cleft skin; LNG: middle anterior lung; MCS: metacarpal skin; NTE: nasal turbinate epithelium; TE: tongue epithelium; NTS: not FMDV targeted sites (MCS, NTE); PIS: persistent infection sites (DNP, DSP); PRS: primary replication sites (DNP, DSP, and LNG); SRS: secondary replication sites (TE, IDC, and CB); -up: up-regulated expression in the tissue group; and -down: down-regulated expression in the tissue group.

### Expression of Integrins αVβ6 and Fibronectin

Integrin β subunits may compete with each other for ITGAV if the expression of ITGAV is a limiting factor. Our results show that the signal intensity of ITGAV was significantly lower than the total of all RGD-binding integrins β (Table 3). Therefore, differential expression of other integrins β besides ITGAV and ITGB6 may also affect the amount of heterodimeric integrins αVβ6 (FMDV receptor). Additionally, the binding between integrins αVβ6 and the ligand, fibronectin (FN1), may hinder the binding of FMDV to the receptors. Therefore, the expressions of integrin αVβ6 heterodimeric receptor and FN1 were examined to determine if the expression of the receptor could explain the mechanisms of FMDV tropism.

**Table 3:** Average expression signal intensity of integrins and fibronectin in the tissues of two infected and three non-infected animals.

Gene	SRS-i	SRS-c	MCS-i	MCS-c	PIS-i	PIS-c	LNG-i	LNG-c	NTE-i	NTE-c
ITGAV	241	134	258	152	279	207	212	176	253	171
ITGA2B	189	184	167	238	177	297	549	548	190	269
ITGAs**	3878	4826	7251	7888	4560	4778	11777	9257	5465	6848
ITGB1	429	297	2164	784	1055	619	3794	2224	1573	799
ITGB3	57*	55*	271	187	555	764	676	654	464	817
ITGB5	199	196	255	120	200	149	140	129	314	184
ITGB6	280*	175	165	115	88	102	151	225	183	168
ITGB8	35*	23*	143	101	93	85	93	85	84	86
ITGBs**	1000*	747*	2998	1307	1990	1719	4931	3414	2618	2054
ITGαVβ6***	68*	33	14	14	12	11	7	12	18	20
FN1	96*	232	595	349	727	842	1592	1437	566	704

MCS: metacarpal skin; LNG: lung; NTE: nasal turbinate epithelium; PIS: persistent infection site; SRS: secondary replication site; -c: not infected; -i: FMDV infected;

* statistically significantly different between SRS and the rests;

** the sum of the signal intensity of ITGB1-binding integrin α subunits (ITGAs) or RGD-binding integrin β subunits (ITGBs) according to cited reference [27]; and ***: estimated level of integrin αVβ6 heterodimeric receptor.

Although the expression of ITGAV was not significantly different among tissues, the SRS expressed higher ITGB6 and lower FN1 than other tissues in infected and non-infected animals (Table 3). The expression of ITGB6 in the SRS increased after FMDV infection, while the expression of FN1 decreased when the infected and non-infected tissues were compared (Table 3). The total expression of RGD-binding integrin β subunits (ITGBs) was the lowest in the SRS of infected and not infected animals (Table 3). Among them, ITGB3 and ITGB8 were DEG in the SRS-down gene set. The estimated level of integrin αVβ6 dimeric receptor was significantly higher in the infected and non-infected SRS than that in other tissues (Table 3). However, the difference was not observed between primary replication sites and the control tissue. Thus, higher number of integrin αVβ6 heterodimeric receptor and lower expression of fibronectin in the SRS especially in the infected animals indicate that the expression levels of FMDV receptor and the ligand are determining factors of FMDV tropism in the secondary infection.

### Bioinformatics of Differential Expression

Because there were less than 5 genes in the NTS-down and PIS-down gene sets, only other six gene sets were used in the bioinformatic analysis to infer biological mechanisms of FMDV tissue tropism. Analysis of the promoter sequences of the DEG identified 33 over-represented transcription factor binding sites (Table 4). Six of these binding sites (V$AP1_Q2_01, V$IRF_Q6, V$IRF2_01, V$ISRE_01, V$STAT_Q6, and V$STAT1_01) are known to play important roles in inducing antiviral innate immune response. V$ISRE_01 (a binding site for transcription factor complex activated by type 1 interferon signaling pathway) was found to be over-represented in the NTS-up, SRS-down, and PIS-up gene sets. V$IRF_Q6 and V$IRF2_01 (binding sites for interferon response factors) were more frequent in the SRS-down gene set, while AP1_Q2_01 (a binding site for transcription factors activated by IL-1cytokine family) appeared more in the SRS-up gene set. V$STAT1_01 (a binding site for STAT1 activated by IFNγ signaling) and V$STAT_Q6 (a site for STATs stimulated by the cytokine receptor-kinase complex) were over-represented in the PRS-up gene set. These results indicate that transcription factors binding to these binding sites may play roles in differential gene expression; therefore, pathways such as interferons and IL-1 cytokines that activate these transcription factors may be involved in determining FMDV tropism.

**Table 4:** Over-represented transcription factor binding sites in the promoters of genes differentially expressed between tissue groups of three non-infected animals.

*Tropism*	*Matrix name*	*Transcription factors*	*Gene set* *
Target versus non- targeted site	V$AP2ALPHA_01	adaptor-related protein complex 2, alpha 1	NTS-up
	V$CRX_Q4	cone-rod homeobox	NTS-up
	V$EBF_Q6	early B-cell factors	NTS-up
	V$ETS_Q6	v-ets erythroblastosis virus E26 oncogene homolog	NTS-up
	V$HLF_01	hepatic leukemia factor	NTS-up
	V$HNF1_Q6	hepatocyte nuclear factor 1	NTS-up
	V$ISRE_01	signal transducer and activator of transcription 1,2, and IRF9	NTS-up
	V$OSF2_Q6	runt-related transcription factor 2	NTS-up
	V$PBX1_02	pre-B-cell leukemia homeobox 1	NTS-up
	V$SPZ1_01	spermatogenic leucine zipper 1	NTS-up
	V$WT1_Q6	Wilms tumor 1	NTS-up
	V$ZNF219_01	zinc finger protein 219	NTS-up
High titer replication	V$CRX_Q4	cone-rod homeobox	SRS-down
and vesicular lesion	V$HNF1_Q6	hepatocyte nuclear factor 1	SRS-down
site	V$IRF_Q6	interferon response factors	SRS-down
	V$IRF2_01	interferon response factor 2	SRS-down
	V$ISRE_01	signal transducer and activator of transcription 1,2, and IRF9	SRS-down
	V$NKX25_Q5	NK2 homeobox 5	SRS-down
	V$PBX1_02	pre-B-cell leukemia homeobox 1	SRS-down
	V$TGIF_01	TGFB-induced factor homeobox 1	SRS-down
	V$VMYB_02	myeloblastosis viral oncogene homolog	SRS-down
	V$AP1_Q2_01	Activating protein-1 transcription factors	SRS-up
	V$COUP_DR1_Q6	paired-like homeodomain transcription factor 2	SRS-up
	V$DR1_Q3	nuclear receptor subfamily 2, group F, member 2	SRS-up
	V$E2A_Q2	transcription factor 3	SRS-up
	V$PPAR_DR1_Q2	peroxisome proliferator-activated receptors	SRS-up
	V$YY1_Q6	YY1 transcription factor	SRS-up
Primary versus non-	V$TGIF_01	TGFB-induced factor homeobox 1	PRS-up
primary replication site	V$STAT_Q6	signal transducer and activator of transcription	PRS-up
	V$STAT1_01	signal transducer and activator of transcription 1	PRS-up
	V$ZIC3_01	Zic family member 3	SRS-up
Persistent versus non-	V$CRX_Q4	cone-rod homeobox	PIS-up
persistent infection site	V$E2F_03	E2F transcription factor	PIS-up
	V$E2F_Q6_01	E2F transcription factor	PIS-up
	V$HNF1_Q6	hepatocyte nuclear factor 1	PIS-up
	V$HSF2_01	heat shock transcription factor 2	PIS-up
	V$IRF_Q6	interferon response factors	PIS-up
	V$ISRE_01	signal transducer and activator of transcription 1,2, and IRF9	PIS-up
	V$NF1_Q6_01	neurofibromin 1	PIS-up
	V$NFY_01	nuclear transcription factor Y	PIS-up
	V$NFY_Q6_01	nuclear transcription factor Y	PIS-up
	V$PBX1_02	pre-B-cell leukemia homeobox 1	PIS-up
	V$SF1_Q6	splicing factor 1	PIS-up
	V$STAT1_01	signal transducer and activator of transcription 1	PIS-up
	V$TGIF_01	TGFB-induced factor homeobox 1	PIS-up
	V$VMYB_02	myeloblastosis viral oncogene homolog	PIS-up

* NTS: not targeted site; PIS: persistent infection site; PRS: primary replication site; SRS: secondary replication site; -up: up-regulated in the groups; and -down: down-regulated in the group -up: up-regulated expression in the tissue group; and -down: down-regulated expression in the tissue group.

Using the ExPlain™ program to identify networks that may be significantly affected by the differential expression, we detected several networks relevant to those identified using the promoter sequences of the DEG and to FMDV infection. Several network key nodes associated with type I interferon signaling were detected in the analysis using the SRS-down (PKR, IFNα, indole-acetaldehyde, Jak1), PRS-down (IFNα), and PRS-up (IFNα, Jak1) gene sets, whereas some involved in IL-1 signaling were found using the SRS-down (p38α, p38β2), PRS-down (p38α), and PRS-up (FOS, IκB-α, IKK-β:IκB-α, IL18, IL1B) gene sets (Table 5). In the SRS-up and SRS-down sets, significant numbers of DEG are in a network regulating uPAR-integrin pathways and a network associated with integrins, which may be relevant to FMDV receptors (Table 5). In the PIS-up gene set, 10 DEG are in a network up-stream of survivin (BRIC5, an inhibitor of apoptosis) and two DEG in the PIS-down gene set were in the down-stream of TNF-alpha regulated network, suggesting the involvement of clearing virus infected cells via inducing cell death. Based on the results of these bioinformatic analyses and their relevance to FMDV infection, we decided to further examine the expression of genes involved in extracellular matrix turnover and IL-1, interferon, and death receptor signaling to determine if the expression could explain the mechanisms of FMDV tropism (biological inferences).

**Table 5:** FMDV receptor- or Immune-related biological networks detected based on the genes differentially expressed between tissue groups of three non-infected animals.

Gene set	Location of DEG	Key nodes of networks	Number of DEG	FDR
SRS-up	down-stream	uPAR:integrin	4	≤0.03
SRS-down	down-stream	Jak2, PKR, TGFB2, TLR4:Btk	≥21	≤0.03
	up-stream	IFNα, indole-acetaldehyde, integrins, Jak1, p38α, p38β2, SOCS-1	≥15	<0.01
PIS-up	down-stream	survivin{ubK48}{ubK63}	10	0.008
PIS-down	up-stream	(TNFα:TNFR1)3:(TRADD:traf2)2:(MEKK1)2	2	0.024
PRS-up	down-stream	FOS, IκB-α, IKK-β:IκB-α, IL18, IL1B,	≥27	<0.05
	Up-stream	IFNα, IL-10R, IL-2Rβ, IL-3R, IL-4R, IL-5R, IL-7R, Jak1, RANK	≥28	<0.03
PRS-down	Up-stream	p38α, SOCS-1, IL-7R, IL-4R, IL-2Rβ, IFNα	≥12	<0.05

DEG: differentially expressed genes; FDR: false discover rate produced by ExPlain program; NTS: not targeted site; PIS: persistent infection site; PRS: primary replication site; SRS: secondary replication site; -up: up-regulated expression in the tissue groups; and -down: down-regulated expression in the tissue group.

### Extracellular Matrix (ECM) Turnover

**Figure 1:**
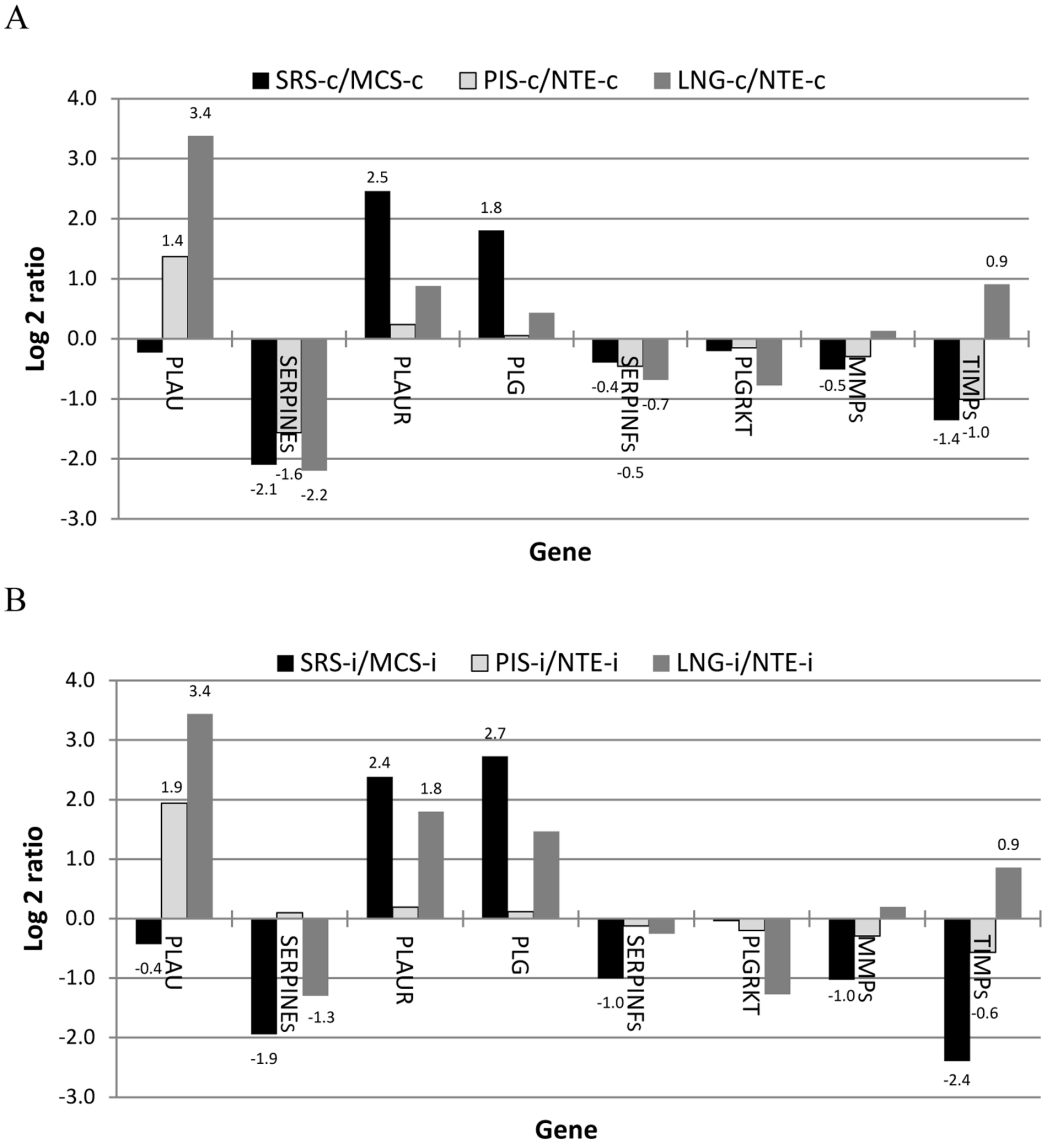
Differential expression of genes regulating extracellular matrix turnover. The log 2 expression ratios (FMDV-targeted tissues versus non-targeted tissue) in three non-infected (A) and two infected (B) animals. MCS: metacarpal skin; LNG: lung; NTE: nasal turbinate epithelium; PIS: persistent infection site; SRS: secondary replication site; -c: not infected; -i: FMDV infected; and data label indicating that the difference between two tissue groups is statistically significant.

Cell attachment involves two types of interactions; cell-ECM and cell-cell adhesion. Cell-ECM interaction is mediated by RGD-binding integrins and regulated by urokinase plasminogen activator receptor (PLAUR or uPAR)-integrin pathways [28]. PLAUR and plasminogen (PLG), the activators of ECM degradation, were expressed significantly higher in the high-titer secondary replication sites, SRS, than those in the negative control tissue (Figure 1A and 1B). On the other hand, SERPINE and SERPINF, the inhibitors of urokinase plasminogen activator (PLAU) and PLG, respectively, were expressed at significantly lower levels in the SRS than those in the control. These differential expressions between the SRS and the control were observed in both infected and non-infected animals. Plasmin can also activate metalloproteases (MMPs) to degrade ECM. Although the average expression of MMPs in the SRS was 1.4 and 2 fold lower than that in the control, the expression of the inhibitors of MMPs (TIMPs) in the control was 2.6 and 5.3 fold higher than that in the SRS in not infected and infected animals, respectively (Figure 1A and 1B). Interestingly, SERPINE and SERPINF were also expressed significantly lower in the non-infected primary replication site, PRS, (3 fold in PIS and 4.6 fold in the lung) than those in the negative control, nasal turbinate epithelium, whereas PLAU, another activator of ECM degradation, was expressed at significantly higher level in both infected and not infected PRS (at least 2.6 fold in PIS and 10.6 fold in the lung) than that in the control (Figure 1A and 1B).

When the expression of these individual genes was normalized to be equally weighted, the SRS expressed significantly higher activators and lower inhibitors of ECM degradation than the negative control tissue, whereas the PRS had significantly lower expression of the inhibitors than their negative control tissue in the not infected animals and the activators were expressed significantly higher in the lung than the control in the infected and non-infected animals. These results indicate that tissues susceptible to primary and secondary infection have gene expression profiles promoting higher ECM turnover.

### IL-1 Gene Family

**Figure 2:**
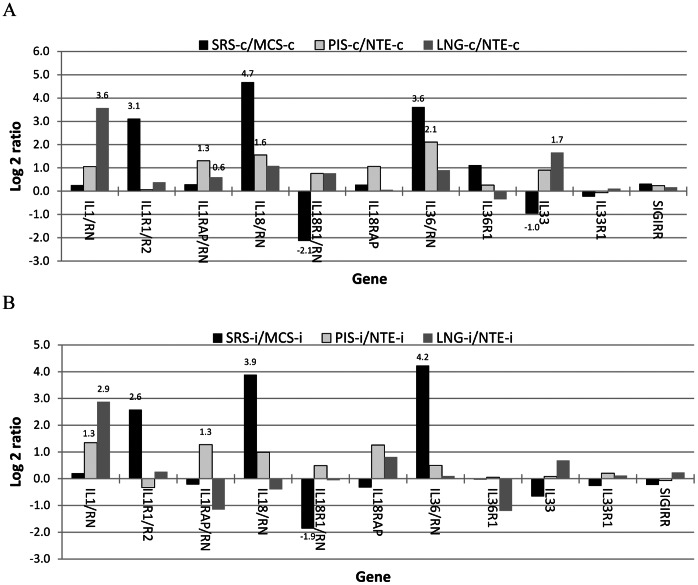
Differential expression of IL-1 family genes. The log 2 expression ratios (FMDV-targeted tissues versus non-target tissue) in three non-infected (A) and two infected (B) animals. MCS: metacarpal skin; LNG: lung; NTE: nasal turbinate epithelium; PIS: persistent infection site; SRS: secondary replication site; -c: not infected; -i: FMDV infected; and data label indicating that the difference between two tissue groups is statistically significant.

In order to evaluate the impact of differential IL-1 cytokine expression, we examined the expression ratios of agonist(s) vs their antagonist(s) instead of the individual genes according to Johnston et al. [29] because there are agonists and antagonist for several receptors and cytokines of the IL-1 gene family [30], [31]. The SRS had significantly higher ratios of IL1R1 vs IL1RII, IL18 vs IL18BP+IL18RN, and IL36 vs IL36RNs but expressed significantly less IL18R1 and IL33 than the negative control, MCS, in the infected and non-infected animals (Figure 2A and 2B). Similarly, primary replication sites had higher IL1 vs IL1RN ratio than their negative control tissue in the infected and non-infected animals (Figure 2A and 2B) and also expressed higher other agonists or have higher agonist vs antagonist ratios than the control in the non-infected animals (Figure 2A). In the infected animals, the differences between the lung and the control, NTE, were smaller and not significant (Figure 2B). When the gene expression was evaluated based on the average Log 2 ratio of targeted vs not targeted tissues, both SRS/MCS and PRS/NTE ratios were at least 1.4 (equivalent to ∼2.6 fold difference between the SRS and MCS) and 0.7 (∼1.6 fold) for IL-1 cytokines in the infected and not infected animals, respectively, and the differences for the receptors were much smaller than those for the cytokines, indicating that susceptibility to FMDV infection in the primary and secondary replication sites was associated with relatively higher local expression of IL-1 agonist cytokines.

### Interferon Signaling

**Figure 3:**
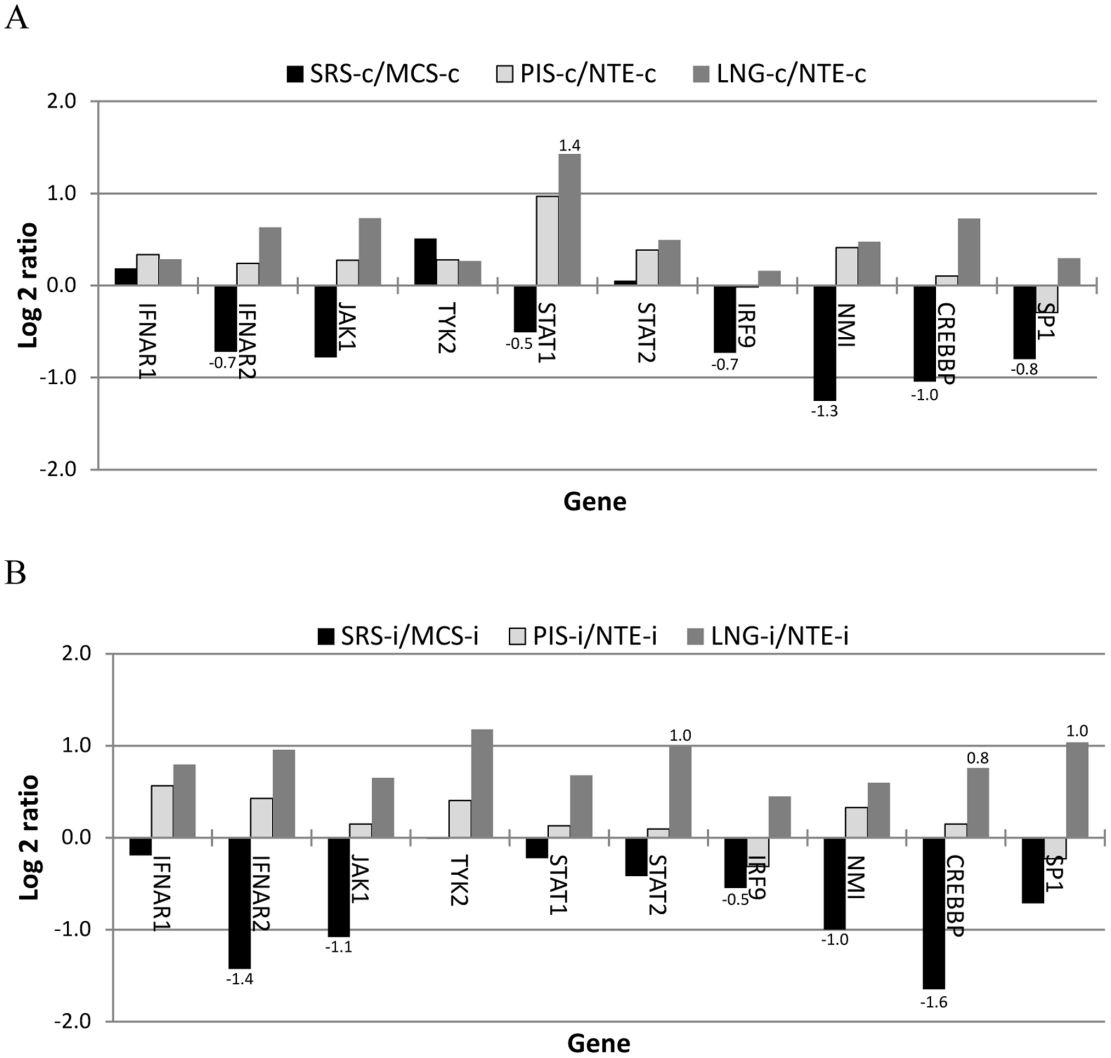
Differential expression of genes in the interferon JAK-STAT signal pathway. The log 2 expression ratios (FMDV-targeted tissues versus non-target tissue) in three non-infected (A) and to infected (B) animals. MCS: metacarpal skin; LNG: lung; NTE: nasal turbinate epithelium; PIS: persistent infection site; SRS: secondary replication site; -c: not infected; -i: FMDV infected; and data label indicating that the difference between two tissue groups is statistically significant.

**Figure 4:**
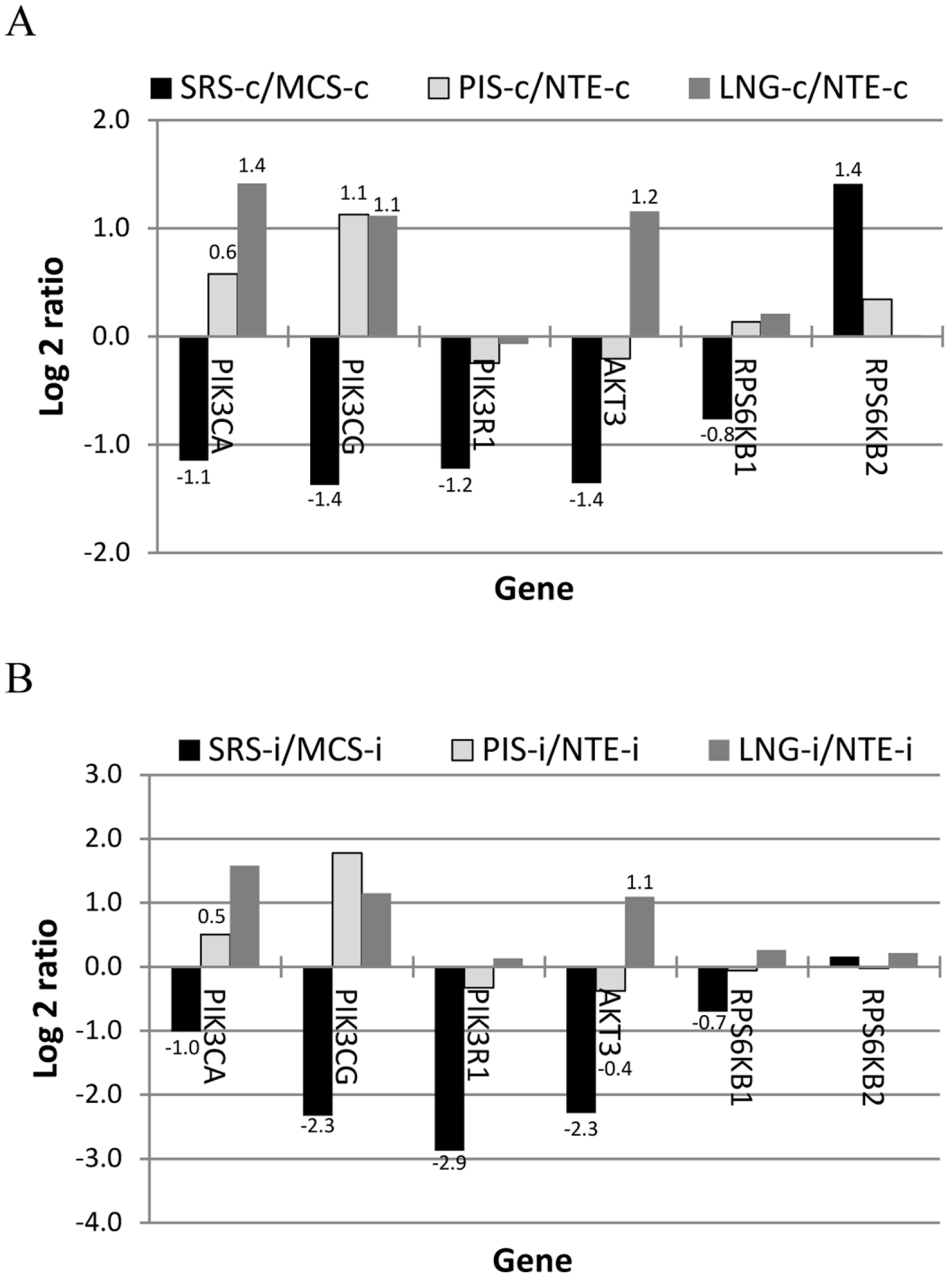
Differential expression of genes in the interferon PI3K-AKT signal pathway. The log 2 expression ratios (FMDV-targeted tissues versus non-target tissue) in three non-infected (A) and two infected (B) animals. MCS: metacarpal skin; LNG: lung; NTE: nasal turbinate epithelium; PIS: persistent infection site; SRS: secondary replication site; -c: not infected; -i: FMDV infected; and data label indicating that the difference between two tissue groups is statistically significant.

We examined the expression of genes in the interferon JAK-STAT and PI3K-AKT pathways (regulating transcription and translation, respectively) according to Kroczynska et al. [32] and found that four of the seven genes in the interferon JAK-STAT signaling pathway were expressed lower in the non-infected SRS than those in the control tissue (three were statistically different) (Figure 3A). Three of four genes known to enhance interferon signing [33] were also expressed significantly lower in the SRS than in the control tissue (Figure 3A). The differential expression was also observed in the infected animals (Figure 3B). The average signal intensity of these genes appeared to be lower in the SRS than the control in the not infected animal but was significantly smaller in the infected tissues. Unlike the SRS, the PRS especially the lung appeared to have higher expression in the genes involved in interferon signaling than their control tissue, NTE (Figure 3). Similarly, differential expression for the genes in the interferon PI3K-AKT signaling pathway was also obtained as those in the JAK-STAT pathway. Five kinases (PIK3CA, PIK3CG, PIK3R1, AKT3, and RPS6KB1) in the pathway were expressed lower in the SRS than those in the control tissue in both infected and non-infected animals. In contrast to the SRS, the expression in the PRS appeared to higher than that in their control tissue (Figure 4A and 4B).

**Figure 5:**
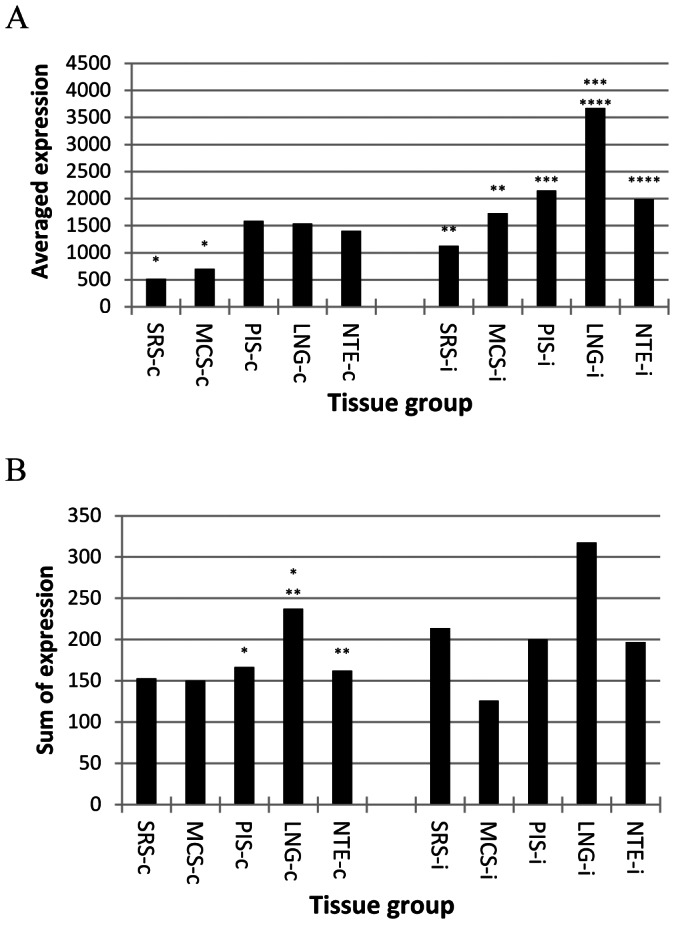
Differential expression of genes of Type I interferons and antiviral effectors. The average signal intensity of interferon-inducible antiviral effectors (A) and the sum of signal intensity of type I interferons (B) in three non-infected and two infected animals. MCS: metacarpal skin; LNG: lung; NTE: nasal turbinate epithelium; PIS: persistent infection site; SRS: secondary replication site; -c: not infected; -i: FMDV infected; and *: statistically significantly different grouped by the number of *.

To compare the status of interferon signaling pathways among tissues, 48 interferon-inducible antiviral effector genes were identified from the Gene Database (http://www.ncbi.nlm.nih.gov/sites/entrez?db=gene) based on the functions discussed by Saddler and Williams [34]. The average expression of these genes was significantly lower in the SRS than that in the control tissue in both infected and not infected animals (Figure 5A). There were no significant differences in overall expression of type I interferon between the SRS and metacarpal skin, though the SRS appeared to have higher type I interferons in the infected animals (Figure 5B), indicating that the differential expression of antiviral effectors between the SRS and the control tissue was mainly due to differences in genes involved in interferon signal transduction.

Unlike the SRS, there were no significant differences in the expression of interferon-inducible antiviral effectors between the PRS and the negative control in the non-infected animals (Figure 5A). However, the lung had higher type I interferon expression than the PIS and negative control (Figure 5B) in the not infected animals and expressed significantly higher antiviral effectors than the PIS and the control in the infected cattle (Figure 5A). As in the non-infected animals, the lung of the infected animals appeared to have higher type I interferon expression than the PIS and the control (Figure 5B), but the differences was not statistically significant. Interferons α and ω in non-infected animals and interferons β in the infected animals accounted for most of the observed differences (data not shown). Taken together with the results of genes involved in the interferon signaling, our results strongly suggest that high-titer FMDV replication and susceptibility to FMDV persistence in primary replication sites are associated with relatively weaker type I interferon signaling when targeted and not targeted tissues were compared.

### Death Receptors and the Ligands

**Figure 6:**
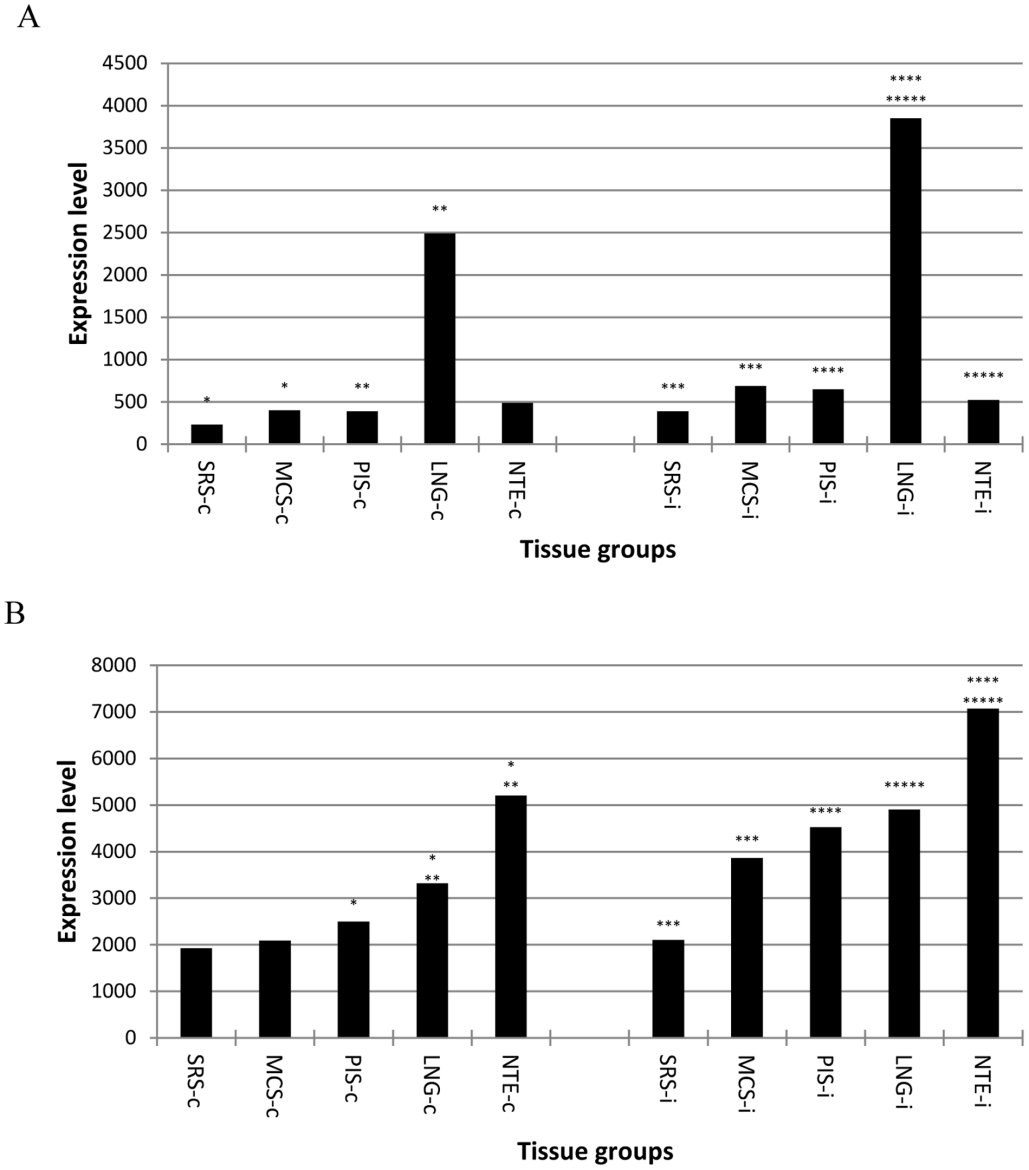
Total expression level of death receptor ligands (A) and death receptors (B) in three non-infected and two infected animals. MCS: metacarpal skin; LNG: lung; NTE: nasal turbinate epithelium; PIS: persistent infection site; SRS: secondary replication site; -c: not infected; -i: FMDV infected; and *: statistically significantly different grouped by the number of *.

It is well known that several members of the TNF super family including LTA, TNF, FASLG, TNFSF10, TNFSF12, and TNFSF15 play important roles in clearing virus by inducing apoptosis and/or necrosis of infected cells via binding to death receptors such as TNFRSF1, FAS, TNFRSF10, TNFRSF21, and TNFRSF25 [35], [36], [37], [38]. There were significant differences in the expression of the death receptors and the ligands between the SRS and the negative control and between the PRS and the control (data not provided). Interestingly, TNFSF10 was the only ligand which expression was significantly induced by FMDV infection in all tissues tested and was significantly higher (5.6 fold in not infected and 7.7 in the infected animals) in the lung than that in those susceptible to persistent infection. When the expression of the receptors and the ligands was added together, the SRS had lower overall expression of death receptor ligands in both infected and not infected animals (Figure 6A) and lower death receptor expression (excluding TNFRSF10 and TNFRSF25, which probes were not in our microarray) in the infected animals (Figure 6B) than the negative control. The overall expression of the ligands in the lung was at least 5 fold higher than that in the PIS and nasal turbinate epithelium of both infected and not infected animals (Figure 6A). There was no difference in the ligand expression between the PIS and nasal turbinate epithelium; however, the PIS had significantly lower overall expression of death receptors tested than the lung and nasal turbinate epithelium in the non-infected cattle and remained lower in the infected animals (Figure 6B). These results suggest that FMDV tropism particularly at persistence sites is associated with relatively lower expression of death receptors and the ligands.

In summary, we found that a large number of genes were differentially expressed between tissues with different FMDV tropism. Analysis of the differentially expressed genes leads to identification of several biological processes that may be affected by the differential expression. Further examination focused on those relevant to FMDV infection according to reported findings shows that FMDV tissue tropism was associated with significantly differential expression in integrin αVβ6 heterodimeric receptor, fibronectin, IL-1 cytokines, death receptors and the ligands, and multiple genes involved in ECM turnover and type I interferon signaling.

## Discussion

FMDV tropism has been known for nearly half a century [39], [40]. However, the molecular basis for FMDV tissue tropism remains mostly unknown. The functional genomics approach used here allows for a systematic investigation of mechanisms involved. Our previous study demonstrated that data obtained with our custom microarray agreed with those measured using real-time PCR [41]. In this study, we used pair-wise two-tissue comparisons instead of tissue group comparisons in order to detect genes with consistent effects across the tissue tested. Various cut-offs ranging from 2 to 1.5 fold difference with *P*< or = 0.05 were tested. We found that the threshold of significantly differential expression at the 1.75 fold was a better cut-off in terms of balancing between false positives and false negatives and between detection power and biological impact of differential expression in order to create gene sets optimal for the pathway/network analysis. Threshold correction for multiple t-tests was not applied because it is too stringent to create the gene sets with adequate gene number for bioinformatic analysis due to diverse tissues within the tissue groups. For example, when we used threshold correction for multiple t-tests according to Leek et al. [42], less than 10 differentially expressed genes were identified (Data not shown).

We used different bioinformatic tools to analyze the gene sets to identify candidate pathways or networks. The results of different bioinformatic analyses support each other, and the results from non-infected animals mostly agree with those from infected animals. Because of substantial intrinsic differences among tissues, we do not expect that all DEG identified play roles in determining FMDV tropism. On the other hand, not all genes involved can be detected with the pair-wise tissue comparisons because differential expression of different genes can have same effect on a biological process as we found in this study. Some of detected biological processes may play roles in determining FMDV tropism but were not recognized in this study due to lack of knowledge. We used an approach based on published biological findings to interpret the gene expression results and focused on those relevant to virus/FMDV infection to make the biological inferences. The examination of gene expression in the detected biological processes revealed that the differential expression found could explain FMDV tropism under the widely accepted assumption of the correlation between gene expression level and the functional activity.

Differential expression in integrins αVβ6, fibronectin, and genes regulating ECM turnover appears to be the major factors determining FMDV tropism based on the magnitude of the differences and the biological process involved. Our results of the estimated expression of integrins αVβ6 alone can only explain in part why SRS is more susceptible to FMDV infection than other tissues, but it cannot elucidate the difference between PRS and the negative control tissue. However, the results of combining the scale of the differential expression and the differentially expressed genes involved in regulating cell-ECM attachment could explain the susceptibility to both primary and secondary infections and the differences between primary and secondary replication sites in terms of virus replication titers and pathogenesis. It has been reported that ECM acts as a barrier against virus spread [43], [44], [45], [46], [47]. Because fibronectin is an ECM protein that binds to integrin αVβ6 receptor with the same RGD sequence motif as FMDV, it is reasonable to hypothesize that higher ECM turnover and the degradation and decreased expression of fibronectin make the receptors more accessible for FMDV to infect cells. Based on the known mechanisms involved in cell-ECM attachment, our results show that all FMDV-susceptible tissues examined here regarding both primary and secondary infection possess similar biological characteristics (high ECM turnover) by differentially expressing the same and/or different genes. Our results could also explain why cells isolated from non-targeted tissues become susceptible to FMDV infection in cell culture and why SRS tissues are susceptible to vesicular lesions caused by several viruses.

Our results show tissue susceptibility to primary and secondary FMDV infection was also associated with relatively higher local expression of IL-1 family agonist genes. IL-1 has been reported to be an important mediator of pemphigus vulgaris [48], which can induce an acantholysis process that resembles vesicular lesions seen in FMD, and the pathogenesis of acantholysis can be mediated via uPA system [49]. More interestingly, it has been reported that high IL-1 level in nasal lavage fluids before virus challenge was associated with the onset of rhinovirus infection in humans [50], indicating high IL-1 level is a susceptible factor of viral respiratory infections. The mechanisms of IL-1 signaling involved in susceptibility to respiratory diseases are unknown. However, IL-1 signaling can induce the expression of the activators of ECM degradation [51], [52], [53]. Therefore, we hypothesize that high expression of IL-1 agonists and high ECM turnover render tissues susceptible to viral infections and vesicular lesions. Our results show that significant differences in the estimated level of integrin αVβ6 heterodimeric receptor and fibronectin expression were observed only in the infected animals, indicating that IL-1 signaling triggered by FMDV infection may also play a key role in this differential expression.

Interferon pretreatment has been known to protect against FMDV infection by inducing expression of interferon-inducible antiviral effectors [54]. The data reported here show that the overall expression of interferon-inducible antiviral effector genes and several genes involved in interferon signaling were significantly lower in the SRS than that in other tissues tested. These results suggest that cells in the SRS tissues are more permissive for viral replication because of their decreased expression of antiviral effectors, which probably due to weaker response to interferon stimulation based on the lower expression of genes involved in the signaling found in this study. This agrees with a previous report showing that type 1 interferon response and the expression level of interferon-stimulated genes were the determining factors of tissue susceptibility to poliovirus infection [55]. The lungs (a primary replication site resistant to FMDV persistent infection) of the infected cattle had higher expression of interferon-inducible antiviral effectors, which was associated with higher expression of type I interferons and genes involved in interferon signaling, than the tissues of primary replication site susceptible to the persistent infection (i.e. pharynx), indicating that interferon signaling may also play roles in determining FMDV persistent infection.

Our bioinformatic analyses also led to the identification of a candidate mechanism that could explain, at least in part, why FMDV targets certain PRS for persistent infection. We have identified all bovine death receptors except TNFRSF10 and have found death domains in these receptors. TNFSF10, also named as tumor necrosis factor-related apoptosis-inducing ligand (TRAIL), has been reported to play critical roles in the clearance of viruses by specifically targeting infected cells to induce apoptosis and/or necrosis and by facilitating cell-mediated cytotoxic immune response [38], [56], [57], [58]. We found that the expression of this cytokine was significantly induced by FMDV infection and was several fold higher in the primary replication site (the lung) resistant to FMDV persistence than that in those susceptible. In addition, there were 10 genes in the survivin network significantly up-regulated in PIS compared to other tissues. Because survivin is an inhibitor of apoptosis [59], up-regulated expressions of genes in this network may render the cells more resistant to apoptosis, which may in turn facilitate persistent infection. Studies conducted by Zhang and Alexandersen [60] and Zhang et al. [61] showed that declining rate of FMDV levels during early infection rather than the virus levels determined FMDV persistent infection. These authors proposed that either variations on the host’s abilities to clear virus or differences in the support of virus replication may determine the establishment of FMDV persistent infection. Our results analyzing death receptor and interferon signaling genes provide molecular evidence supporting both hypotheses.

In conclusion, we have identified differential gene expression at a genomic scale between tissues with different FMDV tropism. FMDV tropism could not be explained by the differential expression of a single gene alone. The bioinformatic analysis of the genes differentially expressed led to identify a few biological processes or pathways. Further examination of genes in those relevant to FMDV infection according to reported findings provide the molecular basis that could explain, at least in part, the mechanisms of why FMDV targets specific tissues for primary, high-titer secondary and persistent infection and causes vesicular lesions. Based on these results, we hypothesize that multiple contributory factors including increased FMDV receptor availability and accessibility resulted from high ECM turnover and differential FMDV receptor and the ligand expression, reduced antiviral innate immunity due to low type I interferon response and/or type I interferon production, and decreased ability to clear infected cells via death receptor signaling play roles in determining FMDV tropism. We also hypothesize that additional increase in already high tissue ECM turnover induced by FMDV infection, likely via triggering the signaling of highly expressed IL-1 cytokines, plays a key role in the pathogenesis of vesicular lesions. This work provides guidance for future research to rationally test these factors and further elucidate the molecular mechanisms of tissue tropism for FMDV. The approaches used in this study may also be applied to understanding tropism of other viral agents.
